# In silico exploration of Red Sea *Bacillus* genomes for natural product biosynthetic gene clusters

**DOI:** 10.1186/s12864-018-4796-5

**Published:** 2018-05-22

**Authors:** Ghofran Othoum, Salim Bougouffa, Rozaimi Razali, Ameerah Bokhari, Soha Alamoudi, André Antunes, Xin Gao, Robert Hoehndorf, Stefan T. Arold, Takashi Gojobori, Heribert Hirt, Ivan Mijakovic, Vladimir B. Bajic, Feras F. Lafi, Magbubah Essack

**Affiliations:** 1 Computational Bioscience Research Center (CBRC), King Abdullah University of Science and Technology (KAUST), Thuwal, 23955-6900 Kingdom of Saudi Arabia; 2 Biological and Environmental Sciences and Engineering Division (BESE), King Abdullah University of Science and Technology (KAUST), Thuwal, 23955-6900 Kingdom of Saudi Arabia; 3 Department of Biology, Science and Arts College, King Abdulaziz University, Rabigh, 21589 Kingdom of Saudi Arabia; 4 Biology Department, Edge Hill University, L39 4QP, Ormskirk, Lancashire UK; 5 Department of Biology and Biological Engineering, Division of Systems & Synthetic Biology, Chalmers University of Technology, Kemivägen 10, 41296 Gothenburg, Sweden; 6 Novo Nordisk Foundation Center for Biosustainability, Technical University of Denmark, 2800 Lyngby, Denmark; 7 Department of Medical Laboratories, Faculty of Health Sciences, American University of Madaba, PO Box 2882, Madaba, Amman JO-11821 Jordan

**Keywords:** *Bacillus licheniformis*, *Bacillus paralicheniformis*, Antimicrobials, Biosynthetic gene clusters, Genome-mining, Nonribosomal peptides, Polyketides, Bacteriocins, Lanthipeptides, Bioinformatics

## Abstract

**Background:**

The increasing spectrum of multidrug-resistant bacteria is a major global public health concern, necessitating discovery of novel antimicrobial agents. Here, members of the genus *Bacillus* are investigated as a potentially attractive source of novel antibiotics due to their broad spectrum of antimicrobial activities. We specifically focus on a computational analysis of the distinctive biosynthetic potential of *Bacillus paralicheniformis* strains isolated from the Red Sea, an ecosystem exposed to adverse, highly saline and hot conditions.

**Results:**

We report the complete circular and annotated genomes of two Red Sea strains, *B. paralicheniformis* Bac48 isolated from mangrove mud and *B. paralicheniformis* Bac84 isolated from microbial mat collected from Rabigh Harbor Lagoon in Saudi Arabia. Comparing the genomes of *B. paralicheniformis* Bac48 and *B. paralicheniformis* Bac84 with nine publicly available complete genomes of *B. licheniformis* and three genomes of *B. paralicheniformis,* revealed that all of the *B*. *paralicheniformis* strains in this study are more enriched in nonribosomal peptides (NRPs). We further report the first computationally identified trans-acyltransferase (trans-AT) nonribosomal peptide synthetase/polyketide synthase (PKS/ NRPS) cluster in strains of this species.

**Conclusions:**

*B. paralicheniformis* species have more genes associated with biosynthesis of antimicrobial bioactive compounds than other previously characterized species of *B. licheniformis*, which suggests that these species are better potential sources for novel antibiotics. Moreover, the genome of the Red Sea strain *B. paralicheniformis* Bac48 is more enriched in modular PKS genes compared to *B. licheniformis* strains and other *B. paralicheniformis* strains. This may be linked to adaptations that strains surviving in the Red Sea underwent to survive in the relatively hot and saline ecosystems.

**Electronic supplementary material:**

The online version of this article (10.1186/s12864-018-4796-5) contains supplementary material, which is available to authorized users.

## Background

*Bacillus licheniformis* is a Gram-positive facultative anaerobe, dubbed an industrial workhorse due to its use in several fields of biotechnology and its ability to secrete large amounts of commercially-used biomolecules and enzymes [1, 2]. These include specialty chemicals (e.g., citric acid and poly-γ-glutamic acids) and enzymes (e.g., proteases and α-amylases used in the food, detergent, textile and paper industries) [3–6]. Most importantly, the antimicrobial capabilities of *B. licheniformis* have been widely reported [7–11] and several *B. licheniformis* strains have been used as biocontrol agents [12–15] (e.g., EcoGuard). Moreover, *B. licheniformis* strains are used in the petroleum industry for microbially enhanced oil recovery [7, 16] due to their ability to produce lipopeptide biosurfactants.

*B. paralicheniformis* is a recently described new species within the *Bacillus* genus [17]. Despite the phylogenetic proximity to *B. licheniformis* that suggests biotechnological relevance, this species remains largely unexplored. The first description of *B. paralicheniformis* showed that it displayed a wider range of antimicrobial capabilities than *B. licheniformis*, despite being unable to produce lichenicidin or bacteriocins as does *B. licheniformis* [18].

A genomic-scale comparison of strains in both species can provide insights into their potential metabolic processes, their biosynthetic capabilities, and their stress adaptations. The evaluation of these properties helps to identify potential industrially relevant strains with novel and/or improved production capabilities of desired compounds [19–22]. One way of assessing the production capabilities of these strains is through the identification of gene clusters that are co-localized in the genome [23]. These biosynthetic gene clusters (BGCs) include nonribosomal peptide synthetases (NRPSs), polyketide synthases (PKSs), and ribosomally synthesized and post-translationally modified peptides (RiPPs) [24].

Ecologically, strains of *B. licheniformis* and *B. paralicheniformis* inhabit diverse environments including marine, freshwater, and food-related niches. This diversification in ecological, and phenotypic properties has led *B. licheniformis* to become one of the most studied *Bacillus* species. Reason being, *Bacillus* strains such as these that are adapted to survive in high osmolarity environments, and have metabolic capacities similar to industrial strains are highly desirable. As in industrial settings, strains are often challenged with increased external osmolarity due to the high-level secretion of metabolites into the growth medium, threatening their productivity, and/or viability [25–27].

An environment that should be explored for such resilient, productive *Bacillus* strains is the Red Sea that exhibits relatively high salinity (36–41 p.s.u), and temperature (24 °C in spring, and up to 35 °C in summer) [28]. It is expected that strains from this environment are able to produce a number of thermo-tolerant enzymes, as well as provide robust microbial cell factories that are able to survive frequent exposure to high salinity and high temperature, and produce sturdier enzymes that might be better suited for industrial applications [29].

In this study, we sequenced and assembled genomes of two *Bacillus* strains, *B. paralicheniformis* Bac48 and *B. paralicheniformis* Bac84, both isolated from the Rabigh Harbor Lagoon of the Red Sea in Saudi Arabia. The reason for this selection has been that we previously reported that antimicrobial activity exhibited by *B. paralicheniformis* Bac84 is more pronounced than *B. paralicheniformis* Bac48, against three-indicator pathogens: *Staphylococcus aureus*, *Salmonella typhimurium*, and *Pseudomonas syringae* [30]*.* In the current study we aimed at studying the relevant differences between these two species in more details. Specifically, we estimated the biosynthetic potential of the two Red Sea strains, along with nine *B. licheniformis* and three *B. paralicheniformis* strains. By grouping identified BGCs into families of gene clusters using genomic similarity, we highlighted the overall unexplored biosynthetic potential of strains from both groups. We further showed the unique presence of putative antimicrobial clusters in the Red Sea strains, focusing on one uniquely structured hybrid PKS/NRPS cluster that was identified in the genome of the *B. paralicheniformis* Bac48.

## Results

### Features of the genomes of the Red Sea *Bacillus* strains

Sequencing the genomes of the Red Sea strains using the SMRT (single molecule real-time) sequencing platform produced 138,867 subreads with a mean length of 9586 bp (298× genome coverage) for *B. paralicheniformis* Bac48 and 108,978 subreads with a mean length of 10,964 bp (273× genome coverage) for *B. paralicheniformis* Bac84 (Additional file 1: Table S1 and Table S2). The assembly produced a single circular chromosome without plasmids for both strains. *B. paralicheniformis* Bac48’s circular chromosome is 4,464,381 bp in length containing 4366 predicted open reading frames (ORFs); 51.5% of the genes are on the positive strand, and 48.5% are on the negative one. *B. paralicheniformis* Bac84’s circular chromosome is 4376,831 bp in length containing 4306 predicted ORFs; 47.8% of genes are on the positive strand and 52.2% are on the negative one. Both genomes have 24 rRNAs and 81 tRNAs genes (Table 1).

**Table 1:** Summary of the genomes and annotation of nine *B. licheniformis* and five *B. paralicheniformis* strains

Strain	GenBank accession number	Genome size (Mb)	N^°^contigs	N^°^ORFs	N^°^ rRNA genes (5S, 16S, 23S)	N^°^ tRNA genes	GC content%	Genomic islands%	Prophage%	Environment	Ref.
*B. paralicheniformis* Bac48	CP023666	4.46	1	4366	24	81	45.87	1.4	2.4	Mangrove mud	–
*B. paralicheniformis* Bac84	CP023665	4.38	1	4306	24	81	45.84	3.3	2.2	Microbial mat	–
*B. licheniformis* DSM13	AE017333.1	4.22	1	4256	21	72	46.19	4.8	6.2	N/A	[2]
*B. licheniformis* HRBL-15TD	CP014781.1	4.25	1	4293	24	81	45.92	5.8	3.6	Fermented food	–
*B. licheniformis* WX-02	CP012110.1	4.29	1	4343	24	79	46.1	6.6	4.4	Soil from Salt Mine	[74]
*B. licheniformis* BL1202	CP017247.1	4.42	1	4533	24	81	46	9	7.6	Soybean paste	–
*B. licheniformis* SCK B11	CP014795.1	4.3	1	4372	24	81	45.93	6.2	6	Korean soybean paste	–
*B. Licheniformis* SCDB 14	CP014842.1 ^a^	4.34	2	4369	24	83	46.27	3.5	2.9	Korean soybean paste	–
	CP014843.1 ^b^										
*B. licheniformis* SCDB 34	CP014793.1	4.48	1	4612	24	81	45.69	12.2	7.9	Korean soybean paste	–
*B. licheniformis* SCCB 37	CP014794.1	4.4	1	4496	24	81	45.96	10	8	Korean soybean paste	–
*B. licheniformis* YNP1-TSU	CM007615.1	4.28	10	4376	6	65	45.87	5.7	4.8	Hot spring	[75]
*B. paralicheniformis* ATCC 9945a	CP005965.1	4.38	1	4392	21	72	45.9	4.8	1.9	Soil	[76]
*B. paralicheniformis* BL-09	CP010524.1	4.39	1	4421	21	72	45.9	2.3	3	Natural fermented sour congee	–
*B. paralicheniformis* MDJK30	CP020352.1	4.35	1	4363	24	81	45.9	2.1	1.6	Rhizosphere	–

^a^Chromosome

^b^Plasmid

**Fig. 1:**
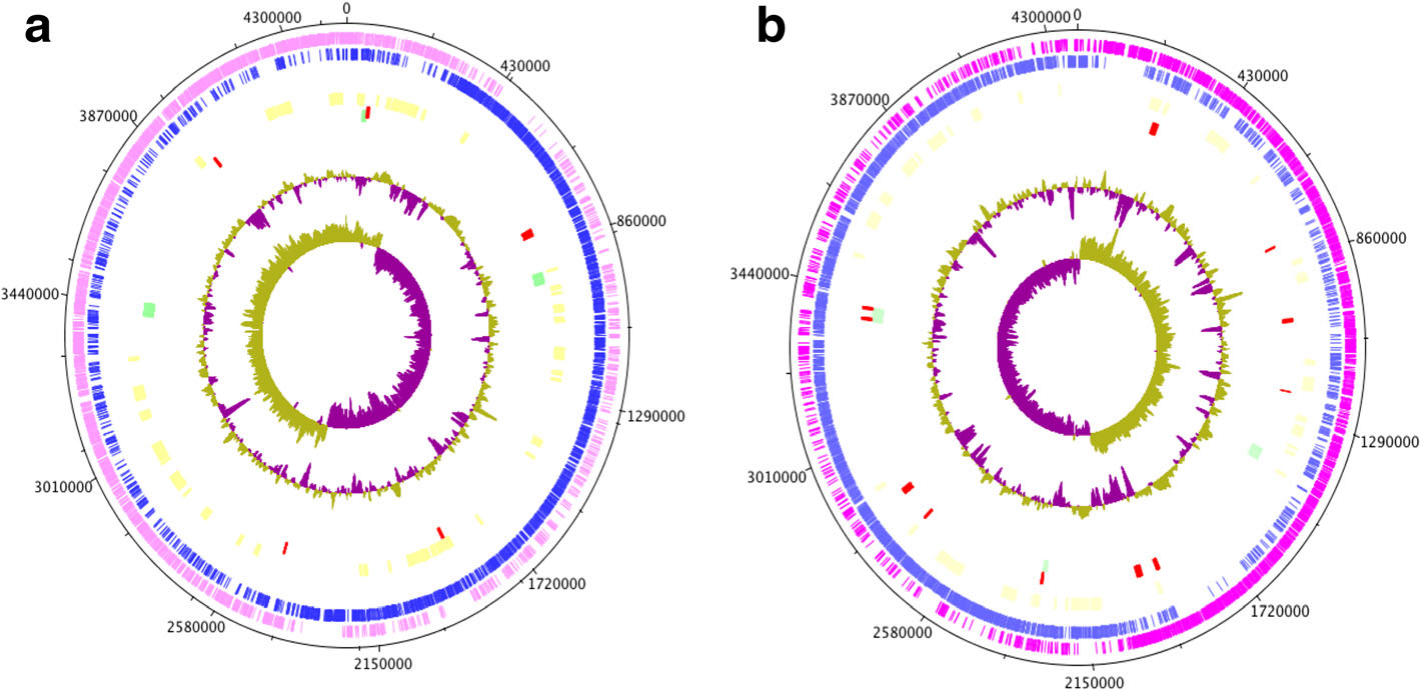
Circular plots of (**a**) *B. paralicheniformis* Bac48 and (**b**) *B. paralicheniformis* Bac84 genomes, showing the distribution of genomic islands, prophages and biosynthetic genes in the genomes**.** The tracks show the following features starting from the outermost track; 1st track (pink): genes on the positive strand; 2nd track (blue): genes on the negative strand; 3rd track (yellow): biosynthetic gene clusters; 4th track (red): horizontally transferred genes; 5th track (green): genes in prophage regions; 6th track: GC-plot where purple and green correspond to below and above average GC content, respectively; 7th track: GC-skew where purple and green correspond to below and above average GC-skew, respectively

Genomic island (GI) prediction identified five GIs in *B. paralicheniformis* Bac48 that include three unique regions (totaling 64.3 Kb and representing 1.4% of the genome) and 14 GIs in *B. paralicheniformis* Bac84 (totaling 142.8 Kb and representing 3.3% of the genome) (Fig. 1, Additional file 1: Table S3). Analysis of prophage sequences in the genome revealed three prophage regions in *B. paralicheniformis* Bac48 (124 genes), with one of them partially overlapping with a GI. Similar analysis in *B. paralicheniformis* Bac84 also identified three prophage regions (121 genes), with two of them partially overlapping with GIs as well (Fig. 1, Additional file 1: Table S4). When compared with the complete genome, the percentage of the genome that constitutes prophages is 2.4% for *B. paralicheniformis* Bac48 and 2.6% for *B. paralicheniformis* Bac84.

These values suggest a reduced number of horizontally transferred elements, and are comparably lower than values in genomes of other industrially important strains such as *B. licheniformis* DSM 13 (where GIs represent 4.8% and prophages represent 6.2% of the genome). This paucity of horizontal gene transfer in *B. paralicheniformis* Bac48 and *B. paralicheniformis* Bac84 genomes is an advantage, as removing GIs and prophages is a necessary step for stabilizing minimized genomes and for streamlining metabolism in biotechnological hosts [31].

### Phylogenetic positioning of the Red Sea *Bacillus* strains

**Fig. 2:**
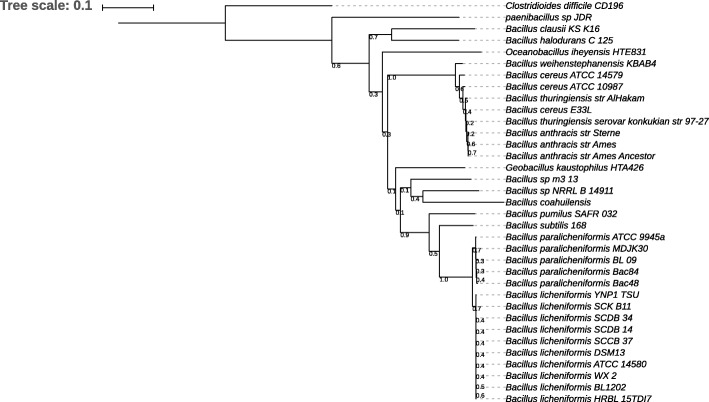
Maximum-likelihood phylogenetic tree of 35 genomes constructed using 494 orthologous groups. *Clostridioides difficile* CD196 was used as the outgroup. Bac48 and Bac84 are placed with the *B. paralicheniformis* subgroup

For a comprehensive comparative analysis of the genomes and to ascertain the phylogenetic position of Bac48 and Bac84 within the *Bacillus* genus, a phylogenetic tree was generated using 494 orthogroups (Fig. 2). According to Wang and Ash [32], phylogenetic trees of *Bacillus* that use this approach are more in line with results from the whole genome feature frequency profiling and are more accurate than phylogenetic trees based on single marker genes such 16S rRNA, *gryB* (gyrase subunit B) or *aroE* (shikimate-5-dehydrogenase) genes.

Other than the two Red Sea strains, our phylogenetic analysis included ten *B. licheniformis* strains, three *B. paralicheniformis* strains and 22 genomes from other representative *Bacilli* [33]. The resulting tree (Fig. 2) shows the phylogenetic proximity of Bac48 and Bac84 to *B. paralicheniformis* strains and reveals them to be more distantly related to *B. licheniformis* than previously reported [30].

### Exploring the biosynthetic potential of *B. paralicheniformis* Bac48 and *B. paralicheniformis* Bac84

To evaluate the biosynthetic potential of the two species (*B. licheniformis* and *B. paralicheniformis*), nine complete *B. licheniformis* and five complete *B. paralicheniformis* genomes, including the two Red Sea strains, were used (Table 1).

**Fig. 3:**
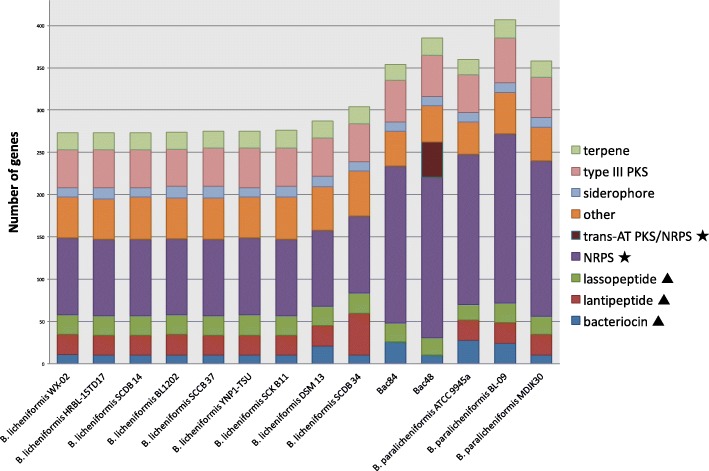
Distribution of genes in biosynthetic gene clusters in nine *B. licheniformis* and five *B. paralicheniformis* genomes. Clusters with modular genes are marked with a star and clusters encoding for ribosomally synthesized peptides are marked with a triangle

On average, each of the analyzed genomes comprised 34 putative biosynthetic gene clusters that were predicted by antiSMASH [24]. These clusters encode peptides/proteins associated with the biosynthesis of one of the following types of secondary metabolites: bacteriocins, lanthipeptides, NRPS, type III PKSs, hybrid PKS/ NRPS clusters and unclassified clusters (Fig. 3). This analysis showed that *B. paralicheniformis* strains have more biosynthetic genes (~ 8.5% of total predicted ORFs) compared to *B. licheniformis* (~ 6.3% of total predicted ORFs). In this study, we focus on two types of compounds that are often associated with high antimicrobial activity: 1/ modular clusters (NRPS and modular PKS), and 2/ ribosomally synthesized peptides, namely modified and unmodified bacteriocins.

**Fig. 4:**
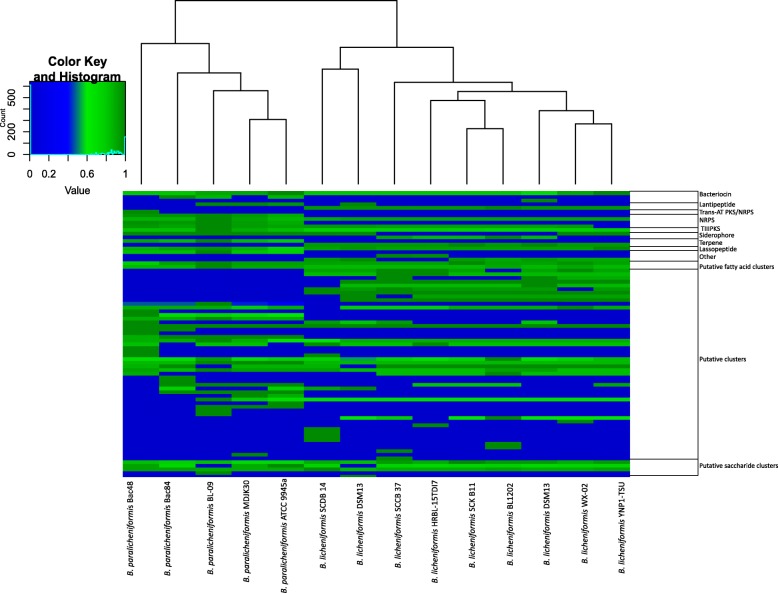
Heat map visualization of the number of genes in BGC groups. There are 54 GCFs with BGCs shared by at least two genomes and 20 BGCs identified to be unique (present in one genome). The number of genes in each GCF is normalized based on the maximum number of genes. Putative clusters are predicted using the ClusterFinder algorithm as implemented in antiSMASH [24]

A total of 480 BGCs were classified into 54 groups (also referred to as gene cluster families GCFs) using scoring similarity networks as implemented in BiG-SCAPE (Fig. 4) [34]. Interestingly, only 6 GCFs (ca. 11% of the total) were assigned to clusters that produce known products or have a similar pathway using threshold similarity of 60% (Additional file 1: Figure S2). This highlights the limited knowledge available for the analyzed strains. Furthermore, these unexplored secondary metabolites can potentially provide new antimicrobial agents and compounds of industrial importance, thus warranting future studies of these BGCs to identify their functions.

### Nonribosomal peptides and modular polyketides

Modular genes in NRPS and PKS clusters are of critical importance when assessing the biotechnological value of strains. Understanding the organization of domains in modules could help advance efforts for the synthesis of products with amended physiochemical properties and enhanced bioactivity [35].

The identified NRPS clusters were grouped into four GCFs with predicted products (Fig. 4). The first group, we found to be conserved across all *B. licheniformis* and *B. paralicheniformis* strains, has 46 genes on average per genome, and shares 46% of its genes with the bacillibactin cluster, a siderophore commonly produced in the *Bacillus* genus [36]. The second GCF of NRPS clusters has 43 genes that include the lichenysin operon (*licABC)*, an efficient biosurfactant from the surfactin family [37–39]. The third and fourth NRPS clusters were only detected in the *B. paralicheniformis* strains*,* including *B. paralicheniformis* Bac48 and *B. paralicheniformis* Bac84, with 50 and 45 genes and with 86 and 100% similarity to the BGC of the antifungal fengycin [40–42] and the narrow-spectrum antibiotic bacitracin [43–46], respectively. In fact, hierarchical clustering shows distinctive presence/absence patterns of BGCs in the two different groups (Fig. 4).

**Fig. 5:**
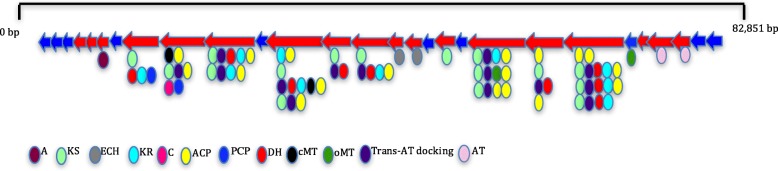
Structure of the hybrid PKS/NRPS cluster present in *B. paralicheniformis* Bac48. Biosynthetic genes are identified with red arrows while non-biosynthetic genes are identified with blue ones. Domains are abbreviated as follows: adenylation (A), ketosynthase (KS), ketoreductase (KR), condensation domain (C), acyl carrier protein (ACP), peptidyl carrier domains (PCP), c-methyltransferase (cMTA), o-methyltransferase (oMT), enoyl-CoA hydratases (ECH), dehydratase (DH), acyltransferase docking site (Trans-AT docking) and acyltransferase (AT)

A hybrid PKS/NRPS cluster was identified in the genome of *B. paralicheniformis* Bac48 (Fig. 5). To the best of our knowledge, this is the first trans-acyltransferase (trans-AT) PKS/NRPS cluster reported in strains of this species. Trans-AT PKS biosynthetic clusters are an emerging class of modular PKSs that are becoming more commonly found in microbial genomes [47]. Structurally, a trans-AT PKS cluster is different from a typical cis-AT PKS in that the AT domain, which loads the substrate onto acyl carrier protein domains (ACPs), is encoded in a separate ORF as independent polypeptide and not integrated into the assembly line [47]. Other trans-AT PKS/NRPS clusters reported within the genus *Bacillus* is the antibiotic bacillaene *pksX* cluster found in *B. subtilis* [48] and the *bae* operon in *B. amyloliquefaciens* [48]. The hybrid trans-AT PKS/NRPS cluster is located 14.6 Kb downstream of a lichenysin synthase operon (*licABC*). The cluster was predicted as a single BGC along with the lichenysin operon; however, due to the large non-biosynthetic gap between the two clusters, the predicted cluster was split into two. The resultant BGC is composed of 29 genes. The cluster extends over 82.8 Kb, which is close in size to the bacillaene and *pksX* cluster (~ 80 Kb) [49]. One of the architectural differences between this cluster and the other trans-AT PKS clusters in *Bacillus* is that there is one NRPS module with its domains (adenylation, condensation and peptidyl carrier domains) extended over two ORFs, while on the other hand, the *bae* cluster has two NRPS modules in two ORFs [49].

The cluster encodes nine multi-domain ORFs, consisting of one adenylation domain (A), 16 ketosynthase domains (KS), ten ketoreductase domains (KR), two peptidyl carrier domains (PCP), 18 acyl carrier protein domains (ACP), nine dehydratase domains (DH), two enoyl-CoA hydratases domains (ECH), two c-methyltransferase domains (cMT), two o-methyltransferase domains (oMT), and one condensation (C) domain. We also identified truncated AT domains that could be used as binding sites for trans-acting AT. The order of the PKS domains and the absence of integrated AT domains in all of the nine PKS/NRPS ORFs in this gene cluster suggest that this is indeed a trans-AT PKS cluster, with two trans-acting AT domains encoded by ORFs that are independent from the polypeptide assembly line. Moreover, the cluster showed similarity to known trans-AT PKSs (71% to elansolid and 57% to thiomarinol) (Additional file 1: Figure S3). Comparing this cluster to known clusters in *Bacillus* revealed a 57% similarity to the bacillaene cluster in *Bacillus amyloliquefaciens* FZB 42. The incomplete homology between the modular genes in this cluster and known clusters in the MIBiG database indicates that the potential active compound synthesized by the trans-AT PKS/NRPS cluster might be a completely novel compound or a compound with similarity in activity to these known compounds. We further identified a putative promoter sequence in the intergenic region upstream of this cluster (Additional file), which strengthens the possible functionality associated with the trans-AT PKS/NRPS cluster.

### Ribosomally synthesized peptides and post-translationally modified peptides (RiPPs): Bacteriocins and lanthipeptide

There is at least one bacteriocin cluster family in each of the analyzed genomes. One of the families was conserved across all the *B. licheniformis and B. paralicheniformis* strains, with an average of nine genes. The clusters in this group had three biosynthetic genes (ribosomal mythelotransferace accessory protein, carbohydrate esterase and an uncharacterized protein) and showed no similarity to any known bacteriocin. Another head-to-tail bacteriocin cluster family was detected in the genomes of *B. paralicheniformis* strains ATCC 9945a, BL-09 and Bac84. Clusters in this family had mostly uncharacterized genes and showed no evident similarity to any known bacteriocin.

Lanthipeptides are a type of bacteriocins that often contain unusual amino acids such as lanthionine and undergo post-translational modification. The fact that these post-translational modification genes are highly conserved assists in the in silico prediction of lanthipeptide clusters [50]. Other features common to lanthipeptide clusters include immunity genes and ABC transporters for bacteriocin export [51].

We found that two-component class II lanthipeptides, in which two peptides processed by a modifying enzyme (*lanM*) [52], are the most common lanthipeptides in the analyzed genomes. *B. licheniformis* strains have three genes mapping to *lchA1*, *lchA2* and *lchM1* in the class II lanthipeptide lichenicidin VK21 cluster. The absence of lichenicidin post-translational modification genes in *B. paralicheniformis* is a distinguishing feature between the two species. A lanthipeptide cluster was detected in the *B. paralicheniformis* genomes (MDJK30, BL-09 and ATCC 9945a), and in *B. licheniformis* SCDB 34 with a mersacidin-like structural gene. The cluster is predicted to be of class II lanthipeptides as it has the *lanM* post-translational modification enzyme. However, other mersacidin genes (*mrsK2*, *mrsR2*, *mrsF*, *mrsG* and *mrsE*) were not detected, indicating that the cluster might be involved in the synthesis of a new product with partial genomic similarity to the genes encoding for the antibiotic mersacidin. No lanthipeptide clusters were predicted in the Red Sea strains; however, the genomes of *B. paralicheniformis* Bac84 harbored a lantibiotic-like cluster, with the subtilin biosynthesis post-translational modification gene *spaB* that encodes the dehydratase of the lanthionine in the subtilin gene cluster (PFAM: PF04738) and subtilin ABC transporter permease (*spaG).* The cluster was not predicted as a lanthipeptide as it lacked other genomic features including the post-translational modification enzyme necessary for the cyclization of lanthionine (*spaC* in the subtilin cluster) and other immunity genes. Additionally, seven genes in the cluster were similar to genes in the rhizocticin biosynthetic cluster, an unusual peptide with antimicrobial activity.

## Discussion

Alignment of the *B. paralicheniformis* Bac48 and *B. paralicheniformis* Bac84 genomes, showed the two genomes to be highly syntenic, except for three large regions present in the *B. paralicheniformis* Bac48 genome that are absent from the *B. paralicheniformis* Bac84 genome (Additional file 1: Figure S1 A and B). The largest non-syntenic block is a ~ 83 Kb region in which the previously described trans-AT PKS/NRPS cluster resides. More specifically, it is worth noting that the trans-AT PKS/NRPS cluster in *B. paralicheniformis* Bac48 has a 27.59% overlap (8 horizontally transferred genes) with a genomic island. Moreover, a bacteriocin cluster composed of 16 genes, has 62.5% overlap with a genomic island in *B. paralicheniformis* Bac84 (10 horizontally transferred genes) (Fig. 1). Obtaining such foreign genes can alter the genotype of a strain through the acquisition of novel metabolic capabilities or altering the existing ones. Herewith allowing strains to adapt/survive in different ecosystems (in this instance, mangrove mud as opposed to microbial mat) [53–55]. This makes the discovery interesting as we previously reported [30] that these strains exhibit different antimicrobial activity; specifically, *B. paralicheniformis* Bac84 has stronger antimicrobial potential against three-indicator pathogens: *Staphylococcus aureus*, *Salmonella typhimurium*, and *Pseudomonas syringae*. Thus, the disparity associated with the antimicrobial activity could be a consequence of the foreign genes providing a novel product with antimicrobial activity.

Also, our analysis showed that the number of NRPS clusters (e.g., lipopeptides) with known predicted products significantly outnumber RiPP clusters with known predicted products, as only lichenicidinVK21 was identified in these clusters. This difference is expected as Firmicutes have been one of the most important sources for the discovery of new lipopeptides, especially as lipopeptides are highlighted to be attractive pharmaceutical or/and industrial products. Investigating the functions of genes in RiPPs showed that, although some of their genes are similar to the ones in known clusters, they are incomplete, with genes absent from the clusters in most of the cases, prevents the use of assigned databases such as MIBiG to determine their final products. Genes in RiPPs from other partially sequenced genomes encode known products such as the recently discovered novel lanthipeptide formicin, produced by *B. paralicheniformis* APC 1576 [56], the bacteriocin bacillocin 490 produced by *B. licheniformis* 490/5 [57] and the bacteriocin-like lichen produced by *B. licheniformis* 26 L-10/3RA [58]. However, *B. paralicheniformis* RiPPs are understudied and the data presented in this in silico analysis highlights the potential for these organisms and the need for further work to validate these findings.

## Conclusion

Several proteins synthesized by *B. licheniformis* strains have high industrial value and are exploited in many applications. However, the bioactive potential of *B. paralicheniformis* species is not completely explored. Here, we report *B. paralicheniformis* strains are more enriched with lipopeptide encoding genes compared to *B. licheniformis* strains. Moreover, the two Red Sea strains, *B. paralicheniformis* Bac48 and *B. paralicheniformis* Bac84, were shown to be more enriched with gene clusters that biosynthesizes bioactive compounds. In spite of the high synteny between the two genomes, we show that *B. paralicheniformis* Bac48 is more enriched in structurally unique modular PKS clusters compared to *B. paralicheniformis* and *B. licheniformis* strains. In future work, more experimental testing is needed in order to exhaustively examine all potential bioactive compounds and the cause of antimicrobial discrepancy between the two strains.

## Method

### Sampling, isolation and purification of bacterial strains

The sampling, isolation and purification of strains Bac48 and Bac84 were previously described by Al-Amoudi et al. (2016) [30]. Both strains were isolated from samples collected from the Rabigh Harbor Lagoon by the Red Sea in Saudi Arabia (39°0′35.762′′E, 22°45′5.582′′ N). Bac48 was isolated from samples that were taken from mangrove mud; while Bac84 was isolated from a microbial mat located 7.5 m away from the lagoon. Eight grams of each sample were homogenized using 10 mL of sterilized Red Sea water at low speed. The supernatant was diluted 5 and 25 folds and plated on media prepared with artificial seawater. Microbial culture containing Bac48 was grown on actinomycetes isolation agar; while culture containing Bac84 was grown on Difco Marine broth 2216 gellan gum media. Inoculated plates were incubated at 28 °C for up to 28 days. Pure colonies were obtained after multiple successful transfers based on morphology then frozen at − 80 °C in ddH_2_O for DNA extraction and 30% glycerol solution for long-term storage.

### DNA extraction and sequencing

Biomass of *B. paralicheniformis* Bac48 and *B. paralicheniformis* Bac84 was obtained after growth under optimal conditions [30]. Genomic DNA was extracted using the Sigma’s GenElute Bacterial Genomic DNA Kit (USA) following the manufacturer’s protocol followed by a second purification step using MO BIO PowerClean Pro Clean-Up Kit (USA). As quality control measures, overnight gel electrophoresis and NanoDrop (Thermo Fisher Scientific, USA) were used to assess purity of DNA, while Qubit 2.0 (Life Technologies, Germany) was used to quantify the DNA. Whole genome sequencing was performed at the Core Lab sequencing facility at KAUST using the PacBio RS II sequencing platform (Pacific Biosciences, USA). The large-insert libraries were sequenced in single-molecule real-time (SMRT) sequencing cells using P6-C4 chemistry.

### Genome assembly

Raw data from PacBio’s RS II were assembled using PacBio’s SMRT Analysis pipeline v2.3.0. using default parameters and genomeSize of 6,000,000 bp, which produced a single contig per library. We visually checked for overlapping ends using Gepard v1.40 [59] which would indicate circular genomes. To circularize both genomes, one end of each contig was trimmed to reduce the amount of overlap, then each contig was split into two halves which were then rejoined using minimus2 [60]. After circularization, multiple rounds of assembly polishing were performed using the SMRT Analysis Resequencing protocol until convergence (Additional file). To assess the quality of the genomes and estimate their completeness and contamination, checkM v1.0.6 [61] taxonomic workflow was used, utilizing single copy genes in the genus *Bacillus*.

### Genome functional annotation and analysis

The complete genome sequences for *B. paralicheniformis* Bac48 and *B. paralicheniformis* Bac84 were annotated using the Automatic Annotation of Microbial Genomes pipeline (AAMG) [62] with default parameters (BLAST bit score of 30) and Prodigal [63] as the chosen gene predictor. For details about the annotation pipeline, tools and databases used, refer to [62].

The overall genome similarities between *B. paralicheniformis* Bac48 and *B. paralicheniformis* Bac84 were inspected using a dot plot that was generated with Gepard v1.40 [59]. Genome variation and synteny were inspected between the two strains using Sibelia v3.0.6 [64]. Prediction of genomic islands was done using IslandViewer v3 [65] and the identification of phage inserts was performed using PHASTER [66]. Finally, circular visualization of the genomes and annotated features were plotted using DNAPlotter [67].

### Strain identification and phylogeny

To build the phylogeny tree, orthologous protein groups (orthogroups) were obtained using OrthoFinder v2.2.1 [68] with default settings. Briefly, an all-vs-all BLASTp analysis [69] was initially performed for the preliminary assignment of gene pairs. Gene pairs were then filtered based on the length-normalized BLAST bitscores to generate a gene pair graph for all-vs-all species. Next, orthogroups were inferred from the graph using the MCL tool v14.137 [70]. After establishing orthology, gene trees were constructed for all orthogroups in the core genomes (all species present) using the alignment-free tool DendroBlast [71] and FastMe v2.1.10 [72]. The Species tree was then reconstructed with support values from the consensus of all gene trees using STAG v1.0.0 (https://github.com/davidemms/STAG) and rooted based on duplication events using STRIDE v1.0.0 [10.1093/molbev/msx259]. We visualized the tree using iTOL (https://itol.embl.de/) [68].

### Biosynthetic gene cluster prediction

Only published strains with complete genomes were included in the analysis to ensure that the identified variations were indeed due to functional differences and not due to the quality of assembly. At the time of our study (May 2017) 12 strains satisfied these requirements, nine *B. licheniformis* and three *B. paralicheniformis*. To avoid potential bias resulting from using different annotation pipelines, all strains were reannotated using the same set of tools and databases.

Biosynthetic and secondary metabolic gene clusters were predicted using antiSMASH v3.0 [24] with the *ClusterFinder* option [23]*.* Additionally, the *KnownClusterBlast* option was used to identify potential products for the clusters from the MIBiG database. Each BLAST hit for the 54 GCFs were manually checked to ensure the similarity accounts for the core biosynthetic genes in the cluster. The promoter prediction tool provided by Softberry [73] was used to predict promoter sequences in the intergenic region upstream of predicted BGCs in the genomes of *B. paralicheniformis* Bac48 and *B. paralicheniformis* Bac84.

## Additional file

Additional file 1: **Table S1.** Basic statistics relating to the PacBio SMRT sequencing that was done for *B. paralicheniformis* B48 and B84. A single SMRT cell was sequenced for each strain. **Table S2.** Levels of completeness and contamination in Bac48 and Bac84 as determined in CheckM. **Figure S1.** Similarity between the genomes of *B. paralicheniformis* Bac48 and *B. paralicheniformis* Bac84. A) Circos figure showing synteny blocks between *B. paralicheniformis* Bac48 and *B. paralicheniformis* Bac84. **Table S3.** List of genomic island regions in the genomes of *B. paralicheniformis* Bac48 and *B. paralicheniformis* Bac84, predicted using IslandViewer [4]. **Table S4.** Predicted prophage regions in *B. paralicheniformis* Bac48 and *B. paralicheniformis* Bac84 and their overlap with GIs. Scores were obtained using PHASTER [5] scoring scheme. Most Common Phage shows the phage ID(s) with the highest number of proteins most similar to proteins in the region. Overlap percentage show the length of overlap region with respect to the length of prophage. **Figure S2.** Similarity network showing 54 groups of similar BGCs. Strains are color coded as per the legend. A product is assigned - shown on top of each group of nodes- if the clusters in the group share more than 60% similarity to the product. Similar gene clusters from different genomes were classified into groups based on homology using BiG-SCAPE [33] and visualized using Cytoscape [6]. (DOCX 4336 kb)

## Abbreviations

A: Adenylation
ACP: Acyl carrier protein
*aroE*: Shikimate-5-dehydrogenase
AT: Acyltransferase
BGC: Biosynthetic gene cluster
C: Condensation
cMT: c-methyltransferase domains
DH: Dehydratase
ECH: Enoyl-CoA hydratase
GCF: Gene cluster family
*gryB*: Gyrase subunit B
KR: Ketoreductase
KS: Ketosynthase
NRPS: Nonribosomal peptide synthetase
oMT: o-methyltransferase
PCP: Peptidyl carrier protein
PKS: Polyketide synthase
RiPP: Ribosomally synthesized and post-translationally modified peptide
SMRT: Single molecule real-time
