# Supplementary material for: In silico exploration of Red Sea Bacillus genomes for natural product biosynthetic gene clusters
Source: BMC Genomics. 2018 May 22;19:382. doi: 10.1186/s12864-018-4796-5 (PMC5964695; doi:10.1186/s12864-018-4796-5)
Supplement: Supplementary file 1 — Table S1. Basic statistics relating to the PacBio SMRT sequencing that was done for B. paralicheniformis B48 and B84. A single SMRT cell was sequenced for each strain. Table S2. Levels of completeness and contamination in Bac48 and Bac84 as determined in CheckM. Figure S1. Similarity between the genomes of B. paralicheniformis Bac48 and B. paralicheniformis Bac84. A) Circos figure showing synteny blocks between B. paralicheniformis Bac48 and B. paralicheniformis Bac84. Table S3. List of genomic island regions in the genomes of B. paralicheniformis Bac48 and B. paralicheniformis Bac84, predicted using IslandViewer [4]. Table S4. Predicted prophage regions in B. paralicheniformis Bac48 and B. paralicheniformis Bac84 and their overlap with GIs. Scores were obtained using PHASTER [5] scoring scheme. Most Common Phage shows the phage ID(s) with the highest number of proteins most similar to proteins in the region. Overlap percentage show the length of overlap region with respect to the length of prophage. Figure S2. Similarity network showing 54 groups of similar BGCs. Strains are color coded as per the legend. A product is assigned - shown on top of each group of nodes- if the clusters in the group share more than 60% similarity to the product. Similar gene clusters from different genomes were classified into groups based on homology using BiG-SCAPE [33] and visualized using Cytoscape [6]. (DOCX 4336 kb) [file 12864_2018_4796_MOESM1_ESM.docx]

*In Silico* Exploration of Red Sea *Bacillus* Genomes for Natural Product Biosynthetic Gene Clusters

Ghofran Othoum ^1^, Salim Bougouffa ^1^, Rozaimi Razali ^1^, Ameerah Bokhari ^2^, Soha Alamoudi ^1^, André Antunes ^3^, Xin Gao ^1^, Robert Hoehndorf ^1^, Stefan T. Arold ^1^, Takashi Gojobori ^1,2^, Heribert Hirt ^2^, Ivan Mijakovic ^4,5^, Vladimir B. Bajic ^1^, Feras F. Lafi ^1,6^, Magbubah Essack ^1#^

^1^ Computational Bioscience Research Center (CBRC), King Abdullah University of Science and Technology (KAUST), Thuwal 23955-6900, Kingdom of Saudi Arabia

^2^ Biological and Environmental Sciences and Engineering Division (BESE), King Abdullah University of Science and Technology (KAUST), Thuwal 23955-6900, Kingdom of Saudi Arabia

^3^ Biology Department, Edge Hill University, Ormskirk, Lancashire L39 4QP, United Kingdom

^4^ Chalmers University of Technology, Division of Systems & Synthetic Biology, Department of Biology and Biological Engineering, Kemivägen 10, 41296 Gothenburg, Sweden

^5^ Novo Nordisk Foundation Center for Biosustainability, Technical University of Denmark, 2800 Lyngby, Denmark

^6^American University of Madaba, Faculty of Health Sciences, Department of Medical Laboratories, Madaba, PO Box 2882, JO-11821 Amman, Jordan

^#^ To whom correspondence should be addressed. Tel: +966 (54) 470 0746; Fax: +966 (2) 802 0127; Email: magbubah.essack@kaust.edu.sa

### Genome Sequencing and Assembly

A Single-Molecule Real-Time (SMRT) cell was run on the PacBio RSII platform in the King Abdullah University of Science and Technology (KAUST) BioCore Lab (Jeddah, Saudi Arabia). Each cell produced 138,867 and 108,978 filtered subread and 1,331,266,339 and 1,194,885,446 bases for *B. paralicheniformis* Bac48 and *B. paralicheniformis* Bac84, respectively (Table S1). *De novo* assembly using the SMRT Analysis pipeline v2.3.0 using the smrtpipe.py commandline script with default parameters and the genomeSize parameter set to 6 Mb. Briefly, the assembly pipeline begins by filtering the SMRT reads (minLength = 50, minSubReadLength = 50, readScore = 0.75), followed by error correction (Minimum Seed Read Length is calculated automatically to produce a minimum coverage of 30x; 22,202 bp minimum read cut-off for *B. paralicheniformis* Bac48 and 22,146 bp for *B. paralicheniformis* Bac84). Assembly of the corrected reads is carried out using the Celera assembler which generates an initial draft assembly followed by a polishing step using the Quiver program. The average read coverage is 298x and 273x for *B. paralicheniformis* Bac48 and *B. paralicheniformis* Bac84, respectively. The assembly produced two contigs for *B. paralicheniformis* Bac48 (4,490,805 and 22,038 bp) and one contig for *B. paralicheniformis* Bac84 (4,400,372 bp). However, the second contig in *B. paralicheniformis* Bac48 had coverage from 2x to 15x and therefore was discarded.

The contigs from the initial assembly were checked for circularization using Gepard [1]. An overlap was observed at peripherals of each contig indicating that both genomes are circular. To circularize, we split each contig at a random location in the middle, then we rejoined the contigs using minimus2 (part of AMOS) [2] producing circular contigs of 4,464,397 bp and 4,376,845 bp for *B. paralicheniformis* Bac48 and *B. paralicheniformis* Bac84, respectively. Finally, each contigs was re-polished using the SMRT Analysis resequencing protocol taking the circularized contigs are the starting reference. The re-polishing step, which uses Quiver, is repeated multiple times taking the polished contig from the previous round as input for the next until convergence. The final genome sizes are 4,464,381 bp and 4,376,831 bp for *B. paralicheniformis* Bac48 and *B. paralicheniformis* Bac84, respectively.

**Table S1:** Basic statistics relating to the PacBio SMRT sequencing that was done for *B. paralicheniformis* B48 and B84. A single SMRT cell was sequenced for each strain.

|  | | ***B. paralicheniformis* Bac48** | ***B. paralicheniformis* Bac84** |
| --- | --- | --- | --- |
| **Polymerase Reads** | **Reads** | 86,252 | 75,932 |
|  | **Bases (bp)** | 1,334,497,375 | 1,197,038,773 |
|  | **N50 (bp)** | 22,785 | 21,104 |
|  | **Average Length (bp)** | 15,472 | 15,764 |
| **Subreads** | **Subreads** | 138,867 | 108,978 |
|  | **Bases** | 1,331,266,339 | 1,194,885,446 |
|  | **N50 (bp)** | 12,578 | 15,215 |
|  | **Average Length (bp)** | 9,586 | 10,964 |

### Assessment of genomes completeness

The completeness and contamination of the Bac48 and Bac84 were evaluated using CheckM (Version1.0.5) [3]. We specifically used the taxonomic workflow, which looks for gene markers within the *Bacillus* genus.

**Table S2:** Levels of completeness and contamination in Bac48 and Bac84 as determined in CheckM.

|  | **Completeness (%)** | **Contamination** |
| --- | --- | --- |
| ***B. paralicheniformis* Bac48** | 98.03 | 0.05 |
| ***B. paralicheniformis* Bac84** | 98.62 | 0.0 |

**Similarity between the genomes of Bac48 and Bac84**

###

###

###

**Figure S1: Similarity between the genomes of *B. paralicheniformis*** **Bac48 and *B. paralicheniformis*** **Bac84. A) Circos figure showing synteny blocks between *B. paralicheniformis*** **Bac48 and *B. paralicheniformis*** **Bac84**. Regions I, II and III are regions in *B. paralicheniformis* Bac48 that are missing in *B. paralicheniformis* Bac84. The coordinates of the two largest regions are from 1,884,514 to 1,968,250 with a total length of 83,736 bp and from 3,977,600 to 3,997,816 with a total length of 20, 216 bp. B) A dotplot between *B. paralicheniformis* Bac48 and *B. paralicheniformis* Bac84 genomes which shows the high concordance between the two genomes.

### Predicted genomic islands and prophage regions in *B. paralicheniformis* Bac48 and *B. paralicheniformis* Bac84

**Table S3.** List of genomic island regions in the genomes of *B. paralicheniformis* Bac48 and *B. paralicheniformis* Bac84, predicted using IslandViewer [4].

| **Genome** | **Name** | **Start position** | **End position** | **Size (bp)** |
| --- | --- | --- | --- | --- |
| ***B. paralicheniformis* Bac84** | GI_1_ | 249,229 | 268,017 | 18,788 |
|  | GI_2_ | 792,253 | 798,734 | 6,481 |
|  | GI_3_ | 1,003,363 | 1,020,318 | 16,955 |
|  | GI_4_ | 1,012,588 | 1,019,457 | 6,869 |
|  | GI_5_ | 1,223,810 | 1,227,971 | 4,161 |
|  | GI_6_ | 1,917,364 | 1,929,075 | 11,711 |
|  | GI_7_ | 1,973,909 | 1,994,365 | 20,456 |
|  | GI_8_ | 1,986,695 | 1,992,844 | 6,149 |
|  | GI_9_ | 2,300,230 | 2,307,438 | 7,208 |
|  | GI_10_ | 2,727,262 | 2,734,510 | 7,248 |
|  | GI_11_ | 2,823,477 | 2,841,438 | 17,961 |
|  | GI_12_ | 3,363,336 | 3,370,202 | 6,866 |
|  | GI_13_ | 3,395,245 | 3,403,627 | 8,382 |
|  | GI_14_ | 3,399,935 | 3,403,534 | 3,599 |
| ***B. paralicheniformis* Bac48** | GI_A_ | 69,655 | 80,660 | 11,005 |
|  | GI_B_ | 776,075 | 799,315 | 23,240 |
|  | GI_C_ | 1,884,577 | 1,898,632 | 14,055 |
|  | GI_D_ | 2,446,070 | 2,454,030 | 7,960 |
|  | GI_E_ | 3,970,771 | 3,978,833 | 8,062 |

**Table S4:** **Predicted prophage regions in *B. paralicheniformis*** **Bac48 and *B. paralicheniformis*** **Bac84 and their overlap with GIs. Scores were obtained using PHASTER [5] scoring scheme**. Most Common Phage shows the phage ID(s) with the highest number of proteins most similar to proteins in the region. Overlap percentage show the length of overlap region with respect to the length of prophage.

|  | **Size (Kb)** | **Completeness** | **PHASTER Score** | **No. of proteins** | **Region** | **Most common phage** | **Overlapping GI region** | **Size of overlap (Kb)** | **GC %** |
| --- | --- | --- | --- | --- | --- | --- | --- | --- | --- |
| ***B. paralicheniformis*** **Bac48** | 28.1 | Incomplete | 50 | 17 | 55436-83598 | PHAGE_Bacill_G_NC_023719(2) | 55436-80,660 | 25.2 | 40.63 |
|  | 36.2 | Intact | 110 | 46 | 916501-952741 | PHAGE_Brevib_Jimmer1_NC_029104(7) | - | - | 47.35 |
|  | 44 | Intact | 97 | 61 | 3407364-3451392 | PHAGE_Bacill_phi105_NC_004167(33) | - | - | 42.68 |
| ***B. paralicheniformis*** **Bac84** | 36.2 | Intact | 110 | 46 | 1415893-1452151 | PHAGE_Bacill_BalMu_1_NC_030945(8) | - | - | 47.23 |
|  | 25.8 | Incomplete | 50 | 17 | 2279889-2305734 | PHAGE_Bacill_G_NC_023719(2) | 2,300,230-2305734 | 5.5 | 40.97 |
|  | 57.6 | Intact | 97 | 58 | 3349191-3406828 | PHAGE_Bacill_phi105_NC_004167(33) | 3,363,336-3,370,202  3,399,935-3,403,534 | 6.8  3.6 | 43.20 |

###

**Biosynthetic gene clusters in *B. licheniformis* and *B.* *paralicheniformis* genomes**


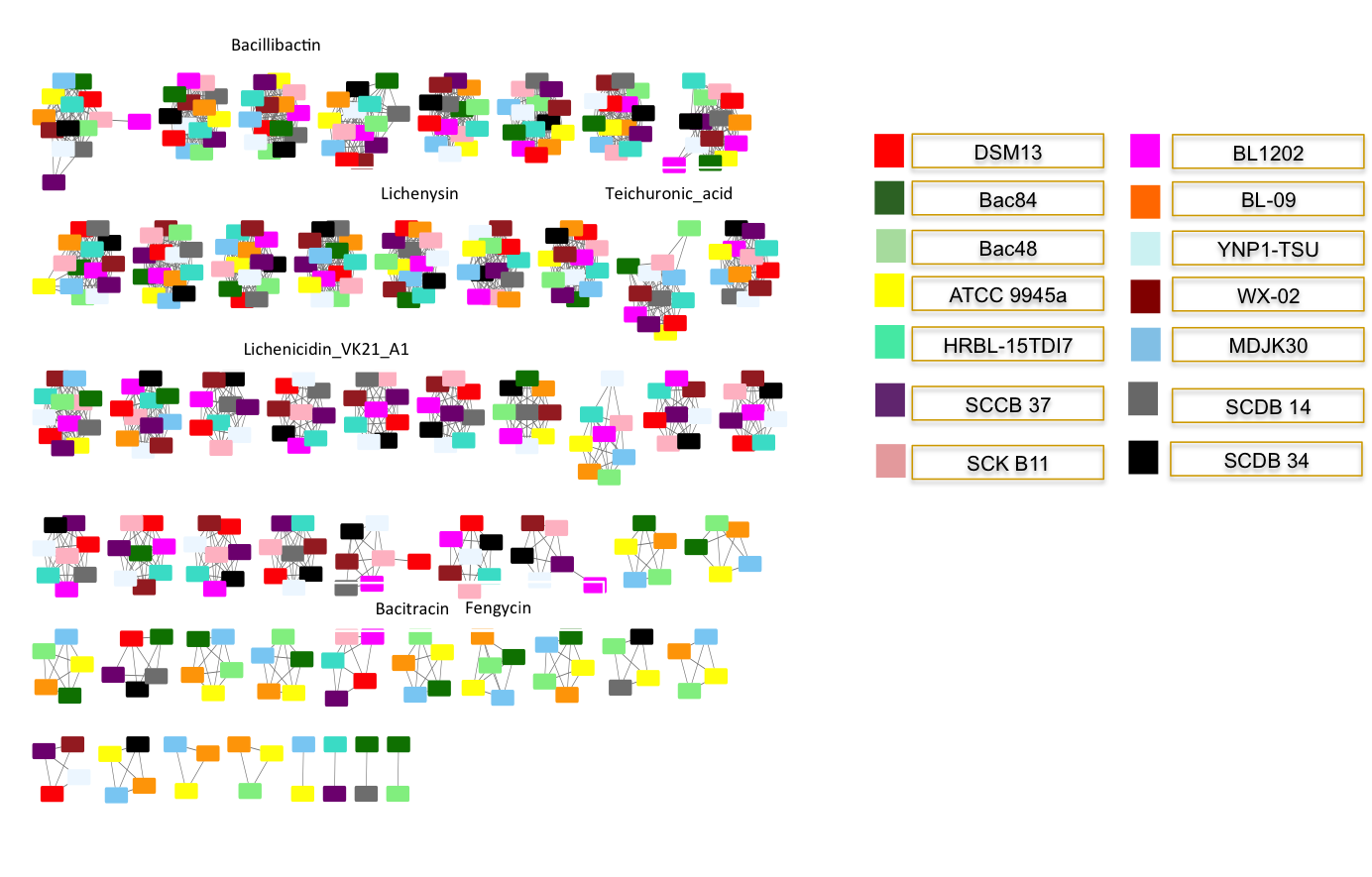


**Figure S2: Similarity network showing 54 groups of similar BGCs.** Strains are color coded as per the legend. A product is assigned - shown on top of each group of nodes- if the clusters in the group share more than 60% similarity to the product. Similar gene clusters from different genomes were classified into groups based on homology using BiG-SCAPE [33] and visualized using Cytoscape [6].


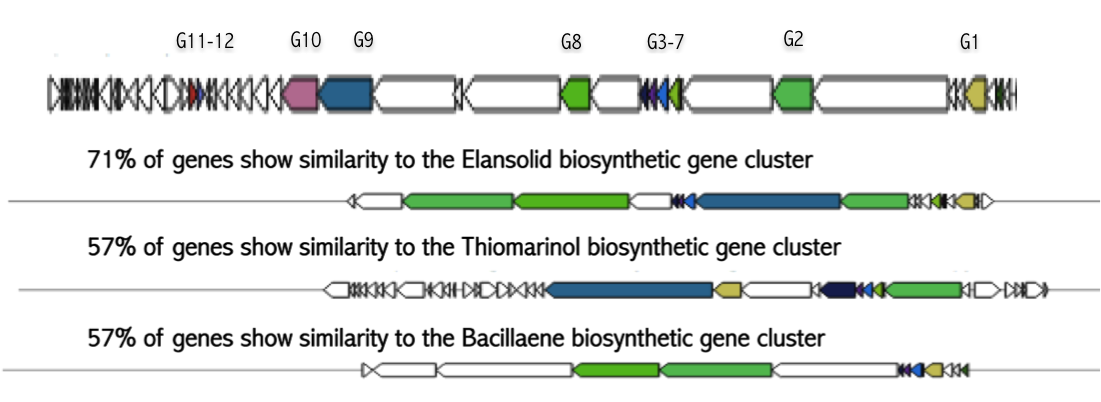


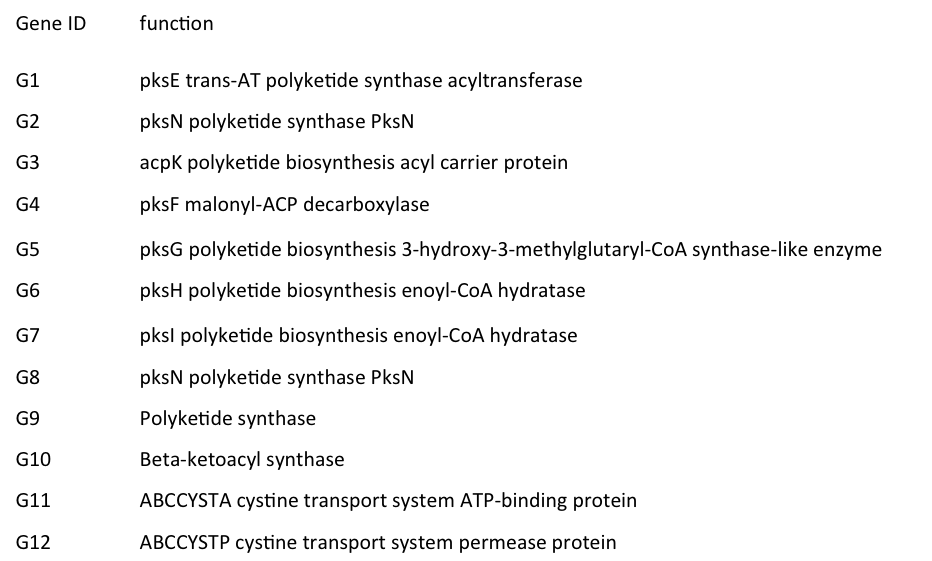


**Promoter sequences upstream of BGCs predicted in the genomes of *B. paralicheniformis* Bac48 and *B. paralicheniformis* Bac84**

Table 1. Predicted promoters for BGCs in the genome of strain B. paralicheniformis Bac48

| **Cluster** | **Number of genes** | **Cluster type** | **Has promoter** |
| --- | --- | --- | --- |
| Cluster 1 | 20 | Terpene | No |
| Cluster 2 | 9 | Putative cluster | No |
| Cluster 3 | 8 | Putative cluster | Yes |
| Cluster 4 | 52 | NRPS | Yes |
| Cluster 5 | 7 | Putative cluster | Yes |
| Cluster 6 | 16 | Putative cluster | No |
| Cluster 7 | 8 | Putative cluster | No |
| Cluster 8 | 18 | Putative cluster | No |
| Cluster 9 | 10 | Putative cluster | Yes |
| Cluster 10 | 22 | Putative fatty acid cluster | No |
| Cluster 11 | 11 | siderophore | No |
| Cluster 12 | 11 | Putative cluster | No |
| Cluster 13 | 18 | Putative fatty acid cluster | No |
| Cluster 14 | 10 | bacteriocin | Yes |
| Cluster 15 | 11 | Putative cluster | Yes |
| Cluster 16 | 16 | Putative saccharide cluster | No |
| Cluster 17 | 8 | Putative cluster | Yes |
| Cluster 18 | 8 | Putative cluster | Yes |
| Cluster 19 | 86 | Trans-AT PKS/NRPS | No |
| Cluster 20 | 5 | Putative cluster | Yes |
| Cluster 21 | 17 | Putative cluster | No |
| Cluster 22 | 9 | Putative cluster | No |
| Cluster 23 | 12 | Putative cluster | No |
| Cluster 24 | 10 | Putative cluster | No |
| Cluster 25 | 21 | lassopeptide | No |
| Cluster 26 | 6 | Putative cluster | No |
| Cluster 27 | 45 | NRPS | Yes |
| Cluster 28 | 7 | Putative cluster | Yes |
| Cluster 29 | 46 | Putative saccharide cluster | Yes |
| Cluster 30 | 28 | Putative saccharide cluster | No |
| Cluster 31 | 43 | Other | No |
| Cluster 32 | 12 | Putative cluster | Yes |
| Cluster 33 | 18 | Putative cluster | Yes |
| Cluster 34 | 48 | NRPS | No |
| Cluster 35 | 49 | T3PKS | No |
| Cluster 36 | 43 | Trans-PKS/NRPS | Yes |

**Table 2**. Predicted promoters for BGCs in the genome of strain B. paralicheniformis Bac84

| **Cluster** | **Number of genes** | **Cluster type** | **Has promoter** |
| --- | --- | --- | --- |
| Cluster 1 | 36 | Putative cluster | No |
| Cluster 2 | 11 | Putative cluster | Yes |
| Cluster 3 | 43 | NRPS | No |
| Cluster 4 | 8 | Putative cluster | No |
| Cluster 5 | 9 | Putative cluster | No |
| Cluster 6 | 15 | Putative saccharide cluster | Yes |
| Cluster 7 | 10 | Bacteriocin | Yes |
| Cluster 8 | 18 | Putative fatty acid cluster | No |
| Cluster 9 | 10 | Putative cluster | Yes |
| Cluster 10 | 11 | Siderophore | No |
| Cluster 11 | 26 | Putative fatty acid cluster | Yes |
| Cluster 12 | 10 | Putative cluster | No |
| Cluster 13 | 13 | Putative cluster | No |
| Cluster 14 | 8 | Putative cluster | No |
| Cluster 15 | 11 | Putative cluster | No |
| Cluster 16 | 52 | NRPS | No |
| Cluster 17 | 7 | Putative cluster | No |
| Cluster 18 | 9 | Putative cluster | No |
| Cluster 19 | 19 | Terpene | No |
| Cluster 20 | 49 | T3PKS | No |
| Cluster 21 | 46 | NRPS | Yes |
| Cluster 22 | 16 | Bacteriocin | No |
| Cluster 23 | 10 | Putative cluster | No |
| Cluster 24 | 11 | Putative cluster | No |
| Cluster 25 | 41 | Other | No |
| Cluster 26 | 28 | Putative saccharide cluster | No |
| Cluster 27 | 37 | Putative saccharide cluster | No |
| Cluster 28 | 8 | Putative cluster | Yes |
| Cluster 29 | 45 | NRPS | Yes |
| Cluster 30 | 6 | Putative cluster | No |
| Cluster 31 | 22 | Lassopeptide | Yes |
| Cluster 32 | 10 | Putative cluster | Yes |
| Cluster 33 | 5 | Putative cluster | Yes |

**References**

1. Krumsiek J, Arnold R, Rattei T: **Gepard: a rapid and sensitive tool for creating dotplots on genome scale**. *Bioinformatics* 2007, **23**(8):1026-1028.

2. Sommer DD, Delcher AL, Salzberg SL, Pop M: **Minimus: a fast, lightweight genome assembler**. *BMC bioinformatics* 2007, **8**(1):64.

3. Parks DH, Imelfort M, Skennerton CT, Hugenholtz P, Tyson GW: **CheckM: assessing the quality of microbial genomes recovered from isolates, single cells, and metagenomes**. *Genome research* 2015, **25**(7):1043-1055.

4. Langille MG, Brinkman FS: **IslandViewer: an integrated interface for computational identification and visualization of genomic islands**. *Bioinformatics* 2009, **25**(5):664-665.

5. Arndt D, Grant JR, Marcu A, Sajed T, Pon A, Liang Y, Wishart DS: **PHASTER: a better, faster version of the PHAST phage search tool**. *Nucleic acids research* 2016, **44**(W1):W16-W21.

6. Smoot ME, Ono K, Ruscheinski J, Wang PL, Ideker T: **Cytoscape 2.8: new features for data integration and network visualization**. *Bioinformatics* 2011, **27**(3):431-432.
